# Airway mucus plugs in asthma and chronic obstructive pulmonary disease: pathobiology, imaging, and implications for clinical trials

**DOI:** 10.1093/ajrccm/aamag309

**Published:** 2026-06-22

**Authors:** Christopher B Bosma, Shawn D Aaron, Bartolome R Celli, Grace Parraga, Sally Seymour, George R Washko, James P Allinson, Federico Baraldi, Richard C Boucher, Mario Castro, Sanjay H Chotirmall, Alejandro A Diaz, John V Fahy, Jonathan G Goldin, Andrew J Halayko, David M G Halpin, Eric Hoffman, Wassim W Labaki, Njira Lugogo, Ella Kazerooni, Mehmet Kesimer, Alberto Papi, Sundaresh Ram, Kathryn A Ramsey, Raúl San José Estépar, Naoya Tanabe, Kyle Whelan, Jadwiga A Wedzicha, Fernando J Martinez, MeiLan K Han

**Affiliations:** Division of Pulmonary and Critical Care, University of Michigan, Ann Arbor, MI, United States; Division of Respirology, Ottawa Hospital Research Institute, University of Ottawa, Ottawa, ON, Canada; Division of Pulmonary and Critical Care Medicine, Department of Medicine, Mass General Brigham and Harvard Medical School, Boston, MA, United States; Robarts Research Institute, Department of Medicine, Department of Medical Biophysics, and Department of Medical Imaging, Western University, London, ON, Canada; Seymour Pharmaceutical Consulting LLC, Washington, DC, United States; Division of Pulmonary and Critical Care Medicine, Department of Medicine, Brigham and Women’s Hospital and Harvard Medical School, Boston, MA, United States; National Heart and Lung Institute, Imperial College London, London, United Kingdom; Department of Translational Medicine, University of Ferrara, Ferrara, Italy; Marisco Lung Institute/Cystic Fibrosis and Pulmonary Research Center, University of North Carolina at Chapel Hill, Chapel Hill, NC, United States; Division of Pulmonary, Critical Care, and Sleep Medicine, University of Kansas Medical Center, Kansas City, KS, United States; Lee Kong Chian School of Medicine, Nanyang Technological University, Singapore; Department of Respiratory and Critical Care Medicine, Tan Tock Seng Hospital, Singapore; Division of Pulmonary and Critical Care Medicine, Department of Medicine, Brigham and Women’s Hospital and Harvard Medical School, Boston, MA, United States; Division of Pulmonary and Critical Care Medicine and the Cardiovascular Research Institute, University of California, San Francisco, San Francisco, CA, United States; Department of Radiology, University of California, Los Angeles, Los Angeles, CA, United States; Department of Internal Medicine, University of Manitoba and Children’s Hospital Research Institute of Manitoba, Winnipeg, Manitoba, Canada; Faculty of Health and Life Sciences, University of Exeter Medical School, University of Exeter, Exeter, United Kingdom; Division of Physiologic Imaging, Department of Radiology, University of Iowa Hospitals and Clinics, Iowa City, IA, United States; Division of Pulmonary and Critical Care, University of Michigan, Ann Arbor, MI, United States; Division of Pulmonary and Critical Care, University of Michigan, Ann Arbor, MI, United States; Department of Radiology, University of Michigan, Ann Arbor, MI, United States; Marisco Lung Institute/Cystic Fibrosis and Pulmonary Research Center, University of North Carolina at Chapel Hill, Chapel Hill, NC, United States; Department of Translational Medicine, University of Ferrara, Ferrara, Italy; Department of Radiology and Imaging Sciences, Department of Biomedical Engineering, Emory University and Georgia Institute of Technology, Atlanta, GA, United States; Wal-yan Respiratory Research Centre, The Kids Research Institute Australia, University of Western Australia, Perth, Australia; Department of Radiology, Brigham and Women’s Hospital and Harvard Medical School, Boston, MA, United States; Department of Respiratory Medicine, Graduate School of Medicine, Kyoto University, Kyoto, Japan; Division of Pulmonary, Allergy, and Critical Care, UMass Memorial Medical Center, Worcester, MA, United States; National Heart and Lung Institute, Imperial College London, London, United Kingdom; Division of Pulmonary, Allergy, and Critical Care, UMass Memorial Medical Center, Worcester, MA, United States; Division of Pulmonary and Critical Care, University of Michigan, Ann Arbor, MI, United States

**Keywords:** mucus plugs, mucus plug score, asthma, COPD

## Abstract

Mucus plugs have been previously recognized as an important pathological feature in asthma and chronic obstructive pulmonary disease (COPD), but their clinical role in these diseases has not been explored in depth until recently. Mucus plug formation is driven by mucus hyperconcentration, changes in mucus viscoelastic properties, impaired clearance, and mucociliary collapse. Scoring systems, such as the bronchopulmonary segment mucus plug score, have been used to associate greater mucus plug burden with poor clinical outcomes. Additional scoring methods obtained through quantitative image processing are currently under development. Mucus plug burden has been associated with greater exacerbations and spirometric decline in both asthma and COPD, as well as greater mortality in COPD. Recently, mucus plug burden has been used as an endpoint in clinical trials to evaluate the effectiveness of biologic therapies in asthma; multiple biologic therapies demonstrated decreases in mucus plug burden and associated improvements in spirometry with treatment. Together, these data suggest that mucus plugs may be a treatable trait in asthma and COPD. Mucus plug burden has current clinical, phenotypic, and predictive utility and shows promise as a future biomarker. Increased incorporation into clinical trials, expanded evidence of treatment effect, and standardization of methodology and imaging protocols will be needed. Computed tomographic detection of mucus plug burden is ready for greater incorporation into both research outcomes and clinical care.

## Introduction

Mucus plugs have been recognized as an important pathologic feature of airway disease since 1819, as described in Laennec’s “Treatise on the Diseases of the Chest and on Mediate Auscultation”.^1^ Although important manuscripts in the 20th century emphasized the role of mucus plug pathology, including work by Hogg and McClean in chronic obstructive pulmonary disease (COPD)^1^^,^^2^ and by Huber and Dunhill in asthma,^3^^,^^4^ the clinical role of mucus plugs in COPD and asthma was not explored in depth until recently. Advances in chest imaging, as well as the development and use of an anatomically based scoring system quantifying mucus plug burden in the lungs, have driven research advances surrounding the clinical significance of mucus plugs.^5^ Scoring provides the field a method to describe clinical traits of patients with high mucus plug burden and explore the efficacy of mucoactive treatments.^5-7^ Subsequently, occlusive mucus plugs have been associated with airflow limitation, disease severity, and even increased mortality in some disease states.^5^^,^^8^^,^^9^ This review describes the emerging role of computed tomography (CT) in detecting airway mucus plugs in asthma and COPD, summarizes evidence from observational cohort studies and clinical trials, and considers future research directions focused on airway mucus plugs.

## Mucus physiology and mucus plug pathophysiology

While other recent reviews have addressed the physiology of mucin and the pathophysiology of mucus plugs in more detail,^10-12^ we provide a concise review as a necessary starting point for understanding the pathophysiology and treatment of mucus plugs in airway disease. Airway mucus is primarily comprised of gel-forming mucins, salt, and water. The principal gel-forming mucins are MUC5AC and MUC5B, which are heavily glycosylated polymers that bind water to create a mucus hydrogel. The hydration of airway mucus is mediated by the relative absorptive (epithelial Na^+^ channel–mediated) versus secretory (CFTR-mediated) ion transport processes of airway epithelia. Mucins are secreted by secretory cells and goblet cells in the airway epithelium and mucus cells in submucosal glands. Secretory cells, goblet cells, mucus cells, and other airway cells (ciliated epithelial cells, serous cells in submucosal gland) secrete antimicrobial and anti-inflammatory proteins that combine with mucins to provide airway mucus with robust host defense properties against inhaled microbes and pollutants. Once formed, airway mucus is cleared by mucociliary clearance and cough.^13^ The concentration and viscoelastic properties of mucus meet the functional needs of transport.^10^

The mechanisms of mucus plug formation are incompletely understood; however, current understanding of mucus pathophysiology can contribute to a greater understanding of clinical implications and to the development of mucoactive treatments. The rheological properties of normal airway mucus indicate a viscoelastic liquid, whereas the properties of pathologic airway mucus indicate a viscoelastic solid.^14-16^ This increase in solid (elastic) behavior reflects increases in mucin concentration and mucin cross-linking,^10^^,^^11^^,^^13^^,^^17^ and results in mucus that is more difficult to transport by the mucociliary elevator. A high concentration of mucins and a high percentage of solids in pathologic mucus result from mucin hypersecretion and mucus dehydration driven by epithelial ion transport.^10^^,^^11^ Cross-linking of mucin polymers, reflecting noncovalent intermucin hydrophobic interactions with globular proteins and perhaps increased covalent disulfide bridges due to inflammation, may generate qualitative changes that contribute to solid gel–like behaviors independent of mucin concentration.^15^^,^^18^^,^^19^ Hyperconcentrated mucus compresses cilia and abolishes effective ciliary beating, allowing direct adhesion of mucus to epithelial surfaces, impaired mucus clearance, and increased plug formation.^20-22^ Compounding mucus-dependent slowing of clearance occur in part due to ciliary pathology resulting from downregulated ciliary genes, including loss and shortening of cilia, as well as reduced ciliary beat frequency.^23^ Once formed, mucus plugs are also hypothesized to contribute to regional hypoxia, which may drive further mucus hyperconcentration, airway injury, and mucus plug persistence.^24^

**Figure 1:**
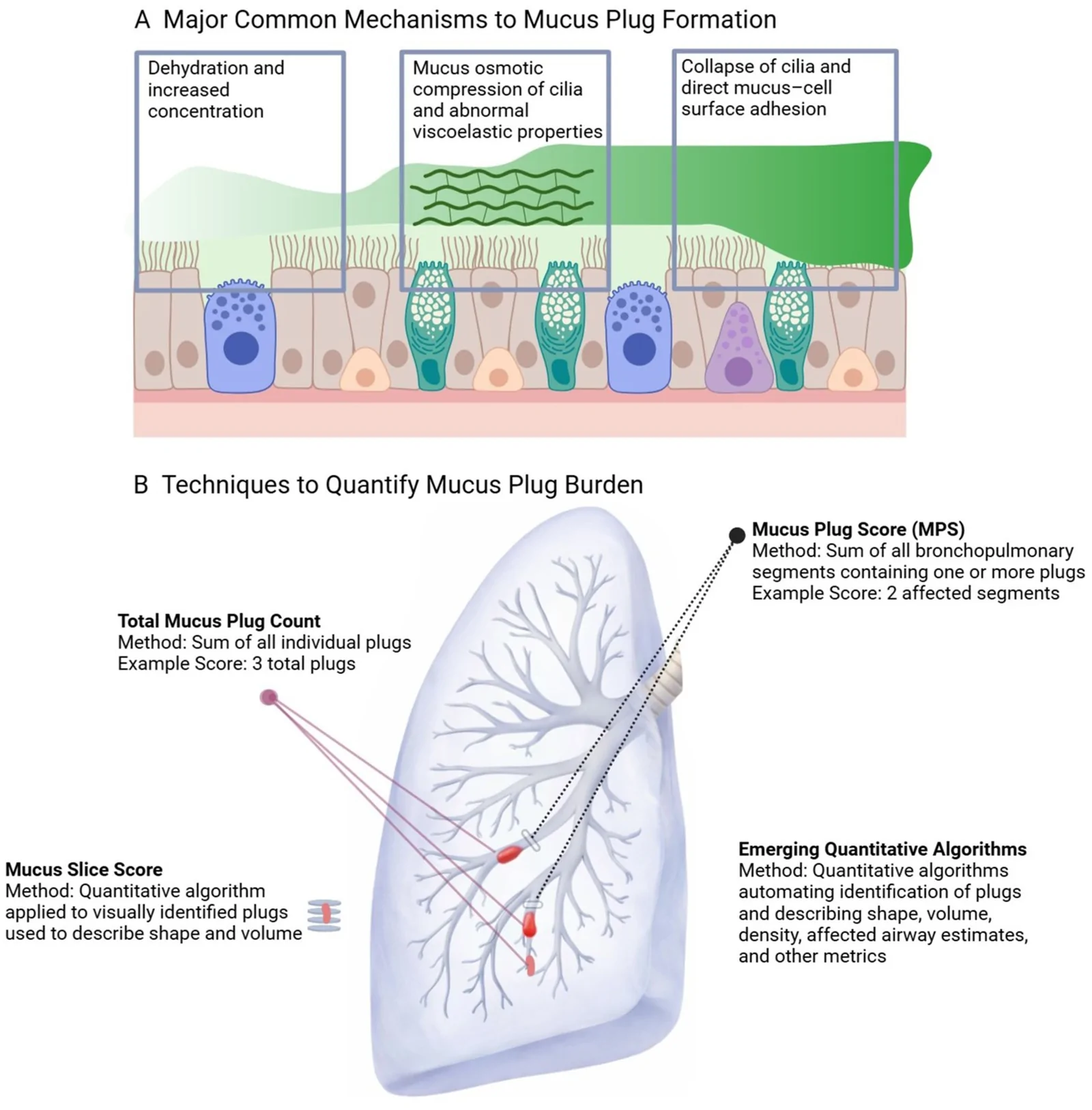
Summary of mucus plug formation and computed tomography scoring methods. (A) The image demonstrates 3 currently considered contributors to mucus plug formation, including increased concentration, viscoelastic solid behavior, and ciliary layer collapse. (B) The image presents currently employed systems to quantify mucus plug burden. Mucus plug score (MPS) has been clinically correlated and validated for visual read. Mucus slice score has been compared to MPS and applies a quantitative algorithm to identified plugs providing information regarding shape and volume. Total mucus plug count includes individual count of every visible plug and is typically conducted by quantitative methods.

While most mucoobstructive diseases share common mechanisms of mucus plug formation and persistence, as displayed in Figure 1, there are also disease-specific expressions of mucus pathology.^13^ For example, in COPD, cigarette smoke is a specific trigger for epithelial cell dysfunction that can result in mucin hypersecretion and reduced mucus hydration.^11^ Specifically, cigarette smoke stimulates production of MUC5B and disproportionately MUC5AC^25^^,^^26^ and has damaging pathologic effects on the function of cilia.^27^ Pathological analysis of mucus plugs in COPD has shown that they often occur in remodeled airways with lymphoid aggregates and are infiltrated by granulocytes.^28^^,^^29^

In asthma, airway remodeling, inflammation of airway walls, and increased mucus secretion are driven by inflammatory cell activity along multiple pathways, including Th2 inflammation, leading to rubbery mucus.^13^ Th2 inflammation is known to upregulate mucin gene expression, particularly MUC5AC, and has been associated with increased mucus plug burden.^5^^,^^30^ Mucus plugs in asthma also occur in remodeled, inflamed airways, in which type 2 immune responses are prominent and the plugs themselves are infiltrated with eosinophils and neutrophils.^13^^,^^29^ In bronchiectasis, mucus plugs are central in disease pathogenesis and exhibit distinctive behavior in proximal and distal bronchiolar disease.^31^ Both regions demonstrate bronchiolar secretory cell features but display distinctions in mucin gene regulation and ectasia.^32^ Hyperconcentrated mucus in bronchiectasis compresses the ­periciliary layer, impacting mucociliary clearance.^33^ Chronic airway inflammation further amplifies this response through interleukin-driven goblet cell hyperplasia and MUC5AC upregulation, while neutrophil-derived interleukins promote MUC5B through NF-κB pathways.^34^^,^^35^ Most significantly in bronchiectasis, but present in multiple disease states, mucus plugs create a nidus for the proliferation and modulation of microbes, which contribute to colonization, chronic infection, and disease progression.^36^^,^^37^

## Respiratory consequences of mucus plugging

### Impact of plug location

The physiologic impact of mucus plugs is partly determined by mucus plug location. Studies have demonstrated that more proximal mucus plugs are associated with greater airflow limitations, as modeled data demonstrate increased resistance and air trapping distal to mucus plugs.^38^ However, the magnitude of mucus plugging in bronchioles (45%-65% of bronchioles plugged in bronchiectasis, primary ciliary dyskinesia, and cystic fibrosis) suggests that distal airways obstruction may also be a significant contributor to airflow obstruction.^31^^,^^39^ Clinical trial data have demonstrated that dissolution of bronchial plugs in study participants with asthma is associated with increased distal airway ventilation.^40^^,^^41^ Whether this association reflects a coordinated clearing of distal and proximal plugs or only renewed ventilation to previously obstructed distal bronchioles is not yet known. In addition to airway generation, mucus plug lobe location may have a physiologic impact. In observational cohort data, preferential burden of mucus plugs in the lower lobes of the lung was ­associated both with lower lung function and increased future respiratory exacerbations.^42^

### Contribution to respiratory failure

In postmortem analyses of lungs from patients who died from an asthma attack, the peripheral airways frequently exhibit luminal occlusion by mucus plugs with inflammatory infiltration.^43^ In patients with severe asthma, widespread airway occlusion is thought to result in alveolar units in which ventilation (V) is severely reduced but blood perfusion (Q) is maintained, leading to very low V/Q ratios and ultimately hypoxemia.^44^ Airway blockage increases respiratory cycle time constants and can lead to air trapping and hyperinflation. Alternatively, poorly ventilated regions distal to mucus plugs may lead to local atelectasis. The same changes occur in patients with COPD,^45^ with more severe consequences, as some of these patients already experience hyperinflation of lungs at baseline due to loss of lung elastic recoil resulting from emphysema.^46^^,^^47^ As a result, the mechanical load of breathing increases, requiring greater intrathoracic pressure to facilitate air movement, which in some patients may negatively impact cardiac output.^48^^,^^49^ During asthma or COPD exacerbations, the increased mucus plug burden may contribute to additional ventilatory and cardiac pathophysiology, both of which may contribute to the mortality associated with these events.^50^^,^^51^

## Detection and characterization of mucus plugs

### Visual and machine learning detection

**Figure 2:**
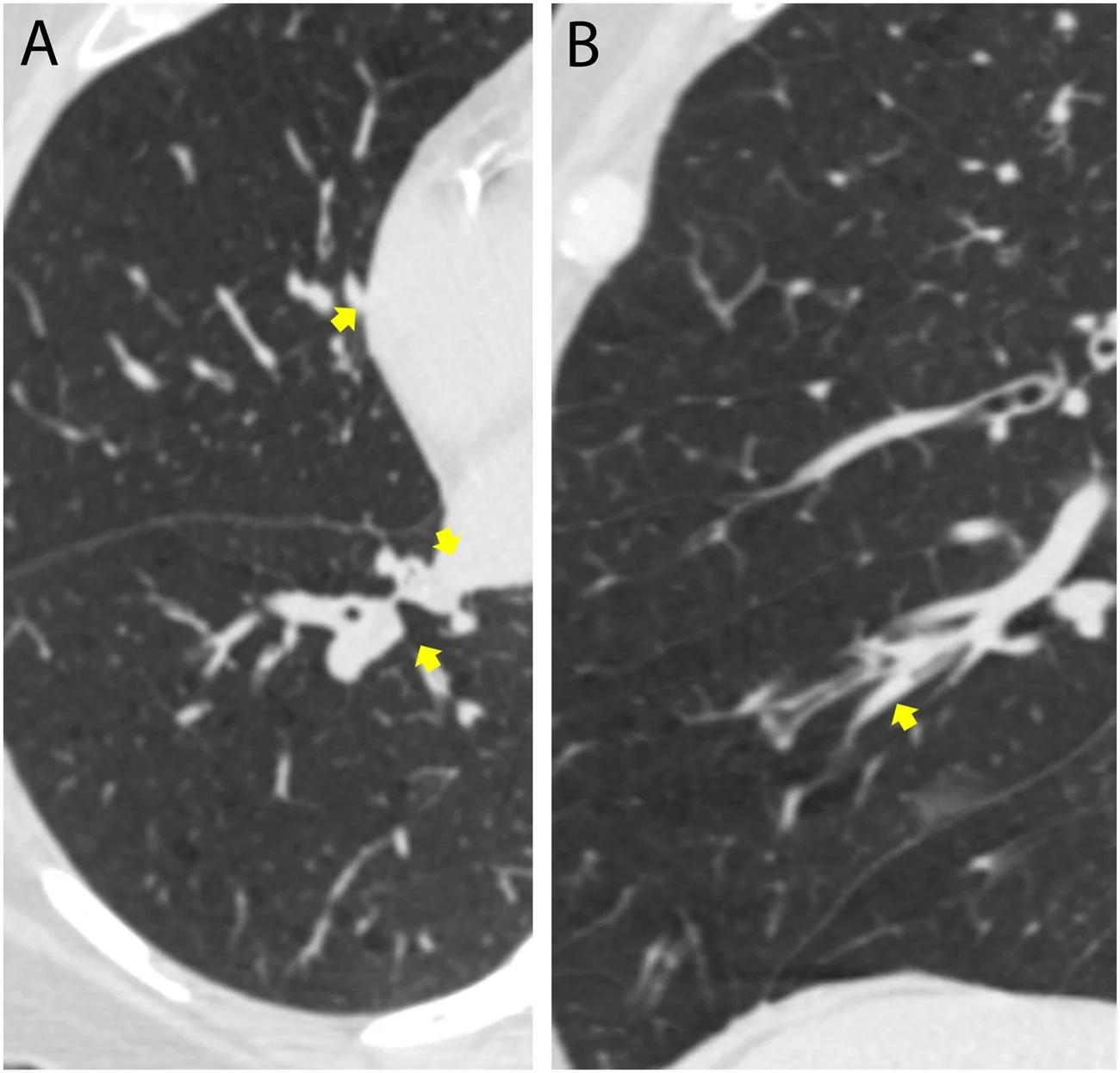
Computed tomography (CT)–detected mucus plugs. Airway-occluding mucus plugs (arrows) on CT images. (A) Axial section of the right middle lobe and right lower lobe. (B) Sagittal section of the right middle lobe of the same individual. Image courtesy of the research team of Dr. Alejandro Diaz.

Mucus plugs can be detected in the small airways both on CT, as displayed in Figure 2, and by magnetic resonance imaging (MRI). The most common definition for a CT-identified mucus plug is an opacity that completely occludes the lumen of an airway. However, some definitions also require visualization of a nonoccluded airway distal to the plug.^5^ Visual identification and quantification of mucus plugs on CT images is time-intensive, as it requires inspection of the airways across all lung segments through several orders of airway branching. Further, there is no universally accepted definition for identifying, classifying, and quantifying mucus plug burden; describing characteristics such as size, length, diameter, degree of airway obstruction; or distinguishing them from other endobronchial filling defects, such as aspirated material or small tumors. Finally, there is no universal agreement on how many orders of small bronchi should be interrogated beyond the segmental airways. In the more peripheral airways, distinguishing airway strictures from narrowing by layering peripheral mucus can be challenging because of their small size, which makes accurate density measurements difficult; yet this may be clinically important.

In recent years, researchers have created machine learning algorithms to expedite the identification of mucus plugs^52-54^ significantly. However, given the lack of agreed-upon visual definitions and paucity of well-annotated CT datasets, the majority of these algorithms have not been validated against visual reads. While some have argued that validation is unnecessary if the algorithm is independently associated with clinical events, concerns remain that identified abnormalities must truly result from plugs rather than from simple airway narrowing, if mucus plugs are to be considered as a therapeutic endpoint. At the moment, there is no consensus on the best visual or machine learning approach for identifying mucus plugs.

### Mucus plug scoring systems

Several visual scoring systems have been proposed to quantify mucus plug burden.^38^ The first and the most common of these scores, the bronchopulmonary segment–based “mucus plug score” (MPS), attributes one point per bronchopulmonary segment containing one or more airway-occluding mucus plugs.^5^^,^^30^^,^^38^ The interreader and intrareader agreement for MPS has been well documented.^8^^,^^9^^,^^55^ Most observational data relating mucus plugs to clinical outcomes has been analyzed using this system. The system provides a score ranging from 0 (no segments affected by mucus plugs) to 18 or 20 (all segments affected), depending on the bronchopulmonary nomenclature used.^5^^,^^8^ This score provides information on the distribution of mucus plugs (segment- and lobe-level) but does not provide information on other characteristics of these plugs (such as length).

**Figure 3:**
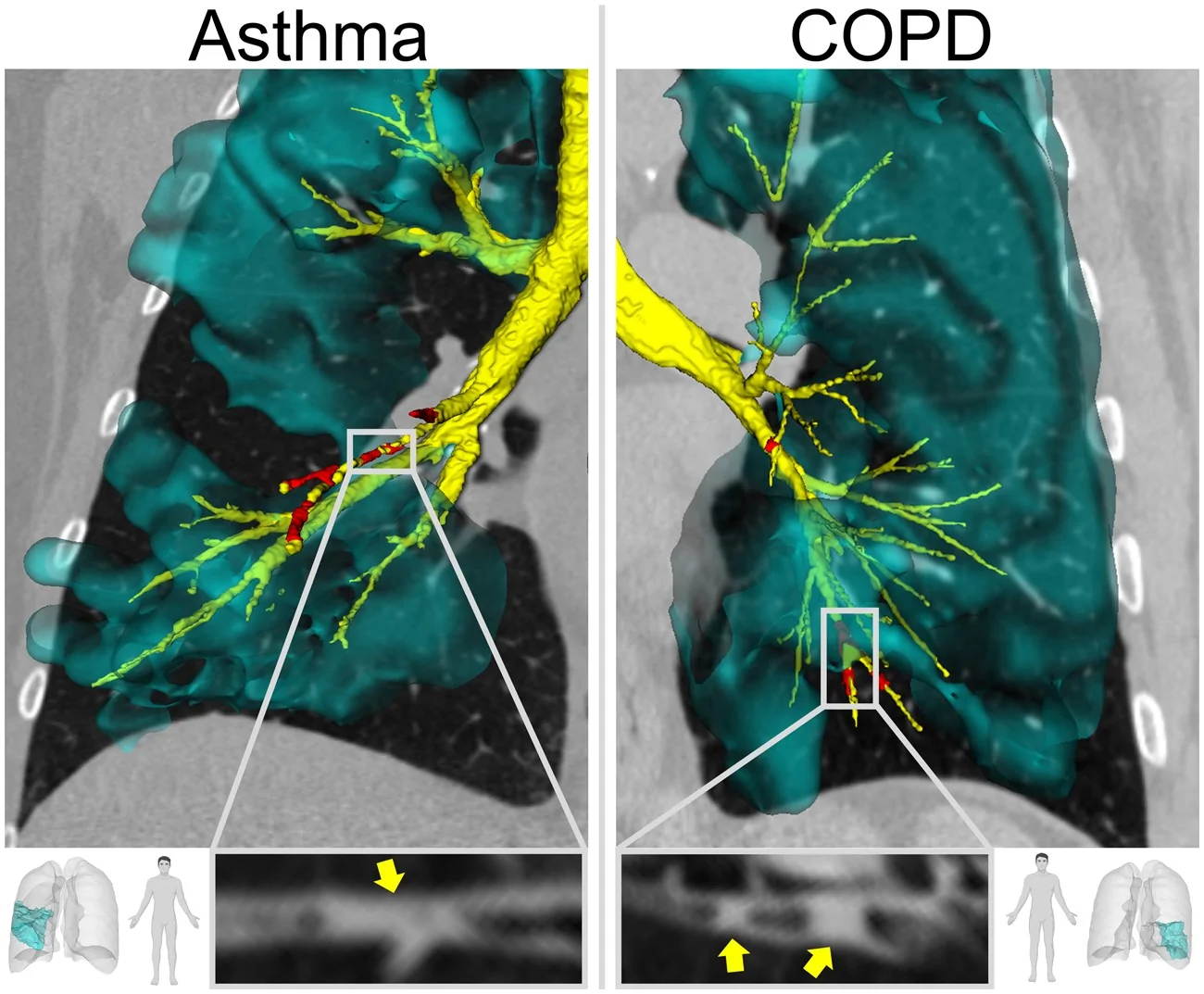
Mucus plug visualization within 3D airway tree. Coronal reconstruction of computed tomography with 3D airway tree (yellow) and center coronal slice ^129^Xe MRI ventilation (cyan) with mucus plug (red) enlarged in inset. Left: Front view of a 59-year-old woman with severe asthma; mucus plug score of 38, mucus plug segment score of 10, prebronchodilator FEV_1_% predicted = 73%, SGRQ score = 75, and VDP = 41%, with a visually obvious ventilation defect in the right middle lobe and “stringy” mucus plug in the lateral segmental bronchus (yellow arrow). Right: Front view of a 63-year-old man with GOLD stage 3 COPD, mucus plug score of 20, mucus plug segment score of 8, prebronchodilator FEV_1_% predicted = 38%, SGRQ score = 77, and VDP = 23%, with a visually obvious ventilation defect in the left lower lobe and “stubby” mucus plug in the lateral segmental bronchus (yellow arrows). Image courtesy of the research team of Grace Parraga, PhD. Abbreviations: COPD, chronic obstructive pulmonary disease; GOLD, Global Initiative for Chronic Obstructive Lung Disease; MRI, magnetic resonance imaging; SGRQ, St. George’s Respiratory Questionnaire; VDP, ventilation defect percentage.

Other scoring systems have been proposed to provide a more detailed description of the size and location of mucus plugs. One study used radiologist annotations of each mucus-occluded airway in axial lung slices from a CT scan. These detailed annotations allowed both a total count of mucus plugs per patient, as well as summation of annotations of occluded airways throughout the lung scan, titled the “mucus slice score” (MSS).^38^ Annotated lung slices can be further analyzed by imaging processing platforms to provide information on mucus plug 3D shape, extent, and spatial distribution within the airway tree. While still relying on radiologist annotation for identification, this method uncovered significant heterogeneity in plug length, with some being short (“stubby”) and others long (“stringy”) as demonstrated in Figure 3.^38^ This subclassification of mucus plugs was associated with differences in plug persistence^38^ and has contributed to further investigations.^56^

Both the MPS and the MSS, in their current use, require an expert visual review of CT lung images, a time-intensive process. Recently, additional scoring methods have been created using artificial intelligence–driven platforms that do not require annotation of every mucus plug by visual reads.^52-54^ These scores can describe total mucus plug counts throughout the entire airway, as well as the location, volume, and additional metrics under investigation. These automated methods have not, however, been validated against visual reads, which is the focus of ongoing research efforts, but they hold promise for making the process of identifying plugs less labor intensive.^52^

### Challenges in mucus plug quantification

All of these approaches have limitations. Distal mucus plugs beyond the eighth generation airways are not captured due to the dose-resolution relationship of current CT imaging. Total airway count has been suggested as a surrogate endpoint for distal mucus plugging, although this could be confounded by airway wall thickening leading to distal airway occlusion.^57^ Additionally, nonocclusive mucus plugs are not included in these scores and have not been studied in detail for clinical associations. Quantifying total mucus volume by summing the cumulative 3D volumes of intraluminal mucus identified within the airway tree on CT is an emerging surrogate measure of overall mucus burden. This measurement provides a continuous, scale-sensitive measure of intraluminal mucus volume^7^; however, the relationship between intraluminal mucus volume and mucus plug burden may not be tightly correlated across all patients. Furthermore, there is growing interest in characteristics of mucus plugs beyond mucus plug count alone. For example, greater mucus plug density measured in Hounsfield units significantly correlates with systemic eosinophilia, perhaps because of greater concentration.^58^

### Magnetic resonance imaging to detect mucus plugs

Chest MRI may help identify mucus plugs and their effects on occluded airways.^59^ However, the extent of research to support this is more limited than for CT. MRI has recently been combined with deep learning models to classify bronchiectasis/wall thickening, and mucus plugs on T2-weighted chest MRI images in patients with cystic fibrosis.^60^ There are concerns regarding the validity of these models in COPD and asthma, as there may be phenotypic differences between plugs between these diseases. MRI has also been used in conjunction with CT images to examine the relationship between mucus plugs and ventilation defects in patients with asthma, as demonstrated in Figure 3.^40^^,^^41^

## Association of mucus plugs with clinical outcomes

In this section, we address the observational evidence linking mucus plugs to poor clinical outcomes in asthma and COPD, along with supporting physiologic and pathologic studies.

### Mucus plugs in asthma

The bronchopulmonary segment–based MPS was initially described and used by the Severe Asthma Research Program (SARP).^5^ The SARP-3 study, which included 136 adults with asthma, demonstrated that patients with asthma had an increased mucus plug burden relative to controls and that mucus plugs could persist in the same airway segment for years. Of interest, mucus plugs often occurred in participants without symptoms of chronic mucus hypersecretion, such as cough and increased sputum production. The study also demonstrated that subjects with mucus plugs in ≥4 segments had severely reduced FEV_1_, and that a high mucus plug burden was associated with systemic and airway eosinophilia, increased fractional exhaled nitric oxide (FeNO) levels, and upregulation of type 2 immune response cytokines in the airways.^5^ Subsequent longitudinal analysis in this cohort demonstrated that persistent mucus plugs (present for >3 years) were associated with increased exacerbations and that the accumulation of additional mucus plugs over time correlated with decreased lung function on spirometry.^61^ These findings have been validated and expanded in other cohorts. Single-site analyses of patients with asthma found that mucus plug burden was associated with lower FEV_1_%, more exacerbations, higher peripheral blood eosinophils, higher total IgE, and elevated FeNO.^62^^,^^63^

In addition to these observational cohorts, small studies have investigated the specific effects of mucus plugs on ventilation and small airway remodeling. A series of 27 patients with asthma, undergoing MRI ventilation studies and CT imaging, noted decreased regional ventilation in patients with mucus plugs.^64^ Histologic analysis of small airways revealed that airways occluded by mucus plugs demonstrated thickened walls and narrow lumens, consistent with airway remodeling.^65^

### Mucus plugs in COPD

Prior to the utilization of CT-detected mucus plugs in observational cohorts, chronic mucus hypersecretion had previously been associated with FEV_1_ decline; in 4427 participants from the United Kingdom aged 20-64 years, chronic mucus hypersecretion, defined as chronic productive cough, was associated with greater FEV_1_ decline and was postulated to represent an early developmental phase of COPD.^66^ Strong pathological evidence from surgically resected lung tissue has shown a direct association of small airway mucus plugs with the severity of COPD.^67^ Furthermore, early work demonstrated that in patients undergoing lung volume reduction surgery for severe emphysema, those who had more small airway mucus plugs in resected tissue had an increased risk of later death.^68^ Subsequent observational cohort studies conducted in COPD Genetic Epidemiology (COPDGene), Evaluation of COPD Longitudinally to Identify Predictive Surrogate Endpoints (ECLIPSE), and the SubPopulations and InteRmediateOutcome Measures In COPD Study (SPIROMICS) have together established that mucus plug burden is associated with increased exacerbations, spirometric decline, and increased mortality in COPD.

A combined cohort analysis of COPDGene and ECLIPSE associated the presence of 1 to 2 and ≥3 visually identified mucus plugs with both current and future respiratory exacerbations in moderate to severe COPD.^69^ Notably, this association was present both in individuals who did not meet spirometric criteria for COPD and in individuals with COPD who did not have symptoms of chronic bronchitis.^70^^,^^71^ A Chinese cohort study of 194 patients corroborated these findings; in multivariate regression analysis, the hazard ratio for moderate to severe COPD exacerbation was 5.02 (95% CI, 1.84-13.75) in individuals with mucus plugs in 4 pulmonary segments compared to those with no mucus plugs.^72^ Initial evidence suggests that mucus plug location may have specific associations with respiratory exacerbations in COPD, with lower lobe–predominant mucus plugs more closely associated with exacerbations.^42^

The relationship between lung function and mucus plugs has been investigated in both large cohorts and in physiologic studies. In the SPIROMICS cohort, MPS was independently associated with lower lung function in individuals who smoke, even when limited emphysema was present, suggesting an independent relationship with lung function apart from emphysema.^47^ Longitudinal analysis of the COPDGene cohort extended this finding, suggesting that participants with persistent and newly formed mucus plugs have accelerated FEV_1_ decline compared to participants with resolving or absent plugs.^73^ Adding to this longitudinal evidence, research has linked mucus plugs to increased airway resistance measured with oscillometry, supporting their obstructive pathophysiologic role in patients with COPD.^74^

Finally, analyses from the COPDGene cohort showed that CT-detected mucus plugs were associated with higher all-cause mortality in participants with COPD.^8^ This finding is corroborated by analyses of 2 cohorts in Japan, which also found increased mortality and airflow obstruction corresponding to MPS.^75^ Interestingly, in addition to risk for exacerbation, more severe disease, FEV_1_ decline, and mortality, initial evidence demonstrates an association between mucus plugs and increased risk for lung cancer in patients with COPD.^44^^,^^76^ Overall, mucus plugs have been associated with a higher risk of both respiratory- and cancer-related deaths in patients with COPD.^77^

As summarized in Table 1, we may conclude that mucus plugging in asthma and COPD is strongly associated with declines in lung function and increased risk of respiratory exacerbations. When combined with pathological and MRI ventilation data, the data suggest that occlusive mucus plugs are likely directly contributing to decreased ventilation in occluded lung areas. In the case of COPD, a greater mucus plug burden has been associated with higher mortality.

**Table 1:** The association of mucus plugs and clinical outcomes in observational studies.

First author	Cohort	Disease state(s)	*N*	Summary of findings
**Dunican^5^**	SARP-3	Asthma Control	168	Initial development of MPS among 168 individuals with asthma or nonasthma controls. MP occurred in greater frequency in patients with asthma than controls, and MP persisted in some individuals in the same segment over years. MPS ≥4 associated with decreased FEV_1_ and increased sputum eosinophils and EPO levels.
**Tang^61^**	SARP-3	Asthma	164	Presence of MP in bronchopulmonary segment on baseline CT associated with MP in same segment at year 3. Presence of MP in a given individuals in baseline CT associated with MP in individual at year 3. Change in MPS was negatively correlated with FEV_1_% predicted.
**Chan^62^**	Single center (Scotland)	Severe asthma	126	MPS score of ≥1 associated with lower FEV_1_%. MPS score of ≥1 associated with greater blood eosinophils, FeNO, and IgE. Participants with more frequent exacerbations were more likely to have MP.
**Tamura^63^**	Single center (Japan)	Asthma COPD ACO	130	Patients with MP had significantly lower FEV_1_% predicted and FVC% predicted. MP associated with IgE and FeNO only in patients with asthma.
**Dunican^47^**	SPIROMICS	COPD Tobacco Exposure Controls	400	MPS associated with lower FEV_1_ and peripheral oxygen saturation. Among tobacco-exposed individuals, MPS associated with worse CAT scores, more frequent exacerbations, and 6MWT.
**Okajima^9^**	COPDGene	COPD Tobacco exposure	500	MPS associated with lower FEV_1_% and SGRQ score. Of subjects with COPD, 73% had persistent luminal plugs over 5 years.
**Wan^69^**	COPDGene ECLIPSE	COPD	4966	Increased MPS (1-2 and ≥3) was associated with increased risk for severe acute exacerbation.
**Borgaonkar^70^**	COPDGene	Tobacco exposure	4474	MPS ≥4 on baseline CT associated with increased exacerbation rates prospectively over follow-up of 12 years.
**Mettler^71^**	COPDGene	COPD	4363	MP present in 627 participants without any symptoms (silent MP). MP without symptoms associated with worse 6MWT, FEV_1_%, and increased odds of severe exacerbation.
**Mettler^73^**	COPDGene	COPD	2118	In participants with 5-year follow-up CT, greater FEV_1_ decline in those with persistent or new MP than in those without MP.
**Diaz^8^**	COPDGene	COPD	4363	MPS ≥1 associated with higher adjusted hazard ratio of mortality.
**Mettler^77^**	COPDGene	COPD	3864	MPS ≥3 associated with higher adjusted hazard ratio of respiratory and cancer deaths.
**Tanabe^75^**	Kyoto-Himeji Hokkaido (Japan)	COPD	295	MPS ≥3 associated with lower FEV_1_%. MPS ≥3 associated with greater mortality in one cohort.
**Li^72^**	Single center (China)	COPD	194	Increasing MPS associated with higher risk of moderate/severe exacerbations. MPS ≥4 associated with exacerbations compared to both MPS of 0 and 1-3.

Abbreviations: 6MWT, 6-minute walk test; ACO, asthma–COPD overlap; CAT, COPD Assessment Test; COPD, chronic obstructive pulmonary disease; COPDGene, Genetic Epidemiology of COPD Cohort; CT, computed tomography; ECLIPSE, Evaluation of COPD Longitudinally to Identify Predictive Surrogate Endpoints; EPO, eosinophil peroxidase; FeNO, fractional exhaled nitric oxide; MP, mucus plug(s), MPS, bronchopulmonary segment–based mucus plug score; SARP-3, Severe Asthma Research Program; SGRQ, St. George’s Respiratory Questionnaire; SPIROMICS, SubPopulations and InteRmediate Outcome Measures in COPD Study.

## Mucus plugs as a treatable trait

Given that mucus plugs are associated with poor clinical outcomes in both asthma and COPD, it is reasonable to review interventional studies (randomized and nonrandomized) that directly or indirectly target the treatment of mucus plugs to attenuate or prevent poor clinical outcomes. Available evidence includes studies that have used mucus plugs as a treatment outcome, secondary analyses, and inferences from large prospective studies.

### Mucus plugs as trial endpoints

Mucus plugs have been used as endpoints in several small observational trials and in 2 randomized controlled clinical trials examining the effectiveness of biologics. Small trials have directly examined dupilumab,^78^ benralizumab,^40^^,^^79^^,^^80^ and mepolizumab^81^^,^^82^ and their effects on the presence of CT-detected mucus plugs. Recently, 2 randomized controlled trials in asthma used mucus plug count as an endpoint. In the VESTIGE trial, 109 patients with moderate to severe asthma aged 21 to 70 years were recruited from multiple countries and randomly assigned to dupilumab or matched placebo for 24 weeks of treatment.^7^ Treatment with dupilumab was associated with increased airway volume and decreased mucus plug scores. In contrast, patients randomized to the placebo group had increased mucus plug scores. Reduction in mucus plug score was associated with improvements in FEV_1_.

In the CASCADE trial, 116 adult patients with asthma who had used medium- or high-dose inhaled corticosteroids (ICS) for the prior year were enrolled and randomized to receive tezepelumab or placebo. A subset of 82 participants had CT scans for evaluation, with a decrease in mucus plug score in the tezepelumab arm.^83^ In a related study, a phase 2a study of an IL-33 monoclonal antibody, tozorakimab, also demonstrated decreased mucus plug scores in 39 patients with COPD who had CT scans at both baseline and 28-week follow-up (although results did not meet statistical significance), and a postbronchodilator, but not prebronchodilator, increase in FEV_1._^84^

### Other treatments targeting mucus plugs

Evidence of the treatment effects of common medications, such as ICS, airway clearance, and mucolytics, on mucus plugs is surprisingly sparse. In the SPIROMICS cohort, mucus plugs were present at the same rate in those treated with and without ICS.^47^^,^^61^ However, a recent study examined the effect of initiating single-inhaler combined ICS/long-acting muscarinic antagonist/long-acting β-agonist (LABA) therapy in 31 patients with moderate to severe asthma who were poorly controlled on ICS/LABA regimens, with follow-up symptom scores, spirometry, ^129^Xe MRI, and CT imaging. In this study, mucus plug occlusion was included as an exploratory outcome and measured at 1 year in 15 participants. This study demonstrated an overall decrease in mucus score among participants treated with single-inhaler triple therapy, as well as improvements in ventilation defect percentage, airway remodeling, and airflow limitation.^85^

### The case for causality

**Figure 4:**
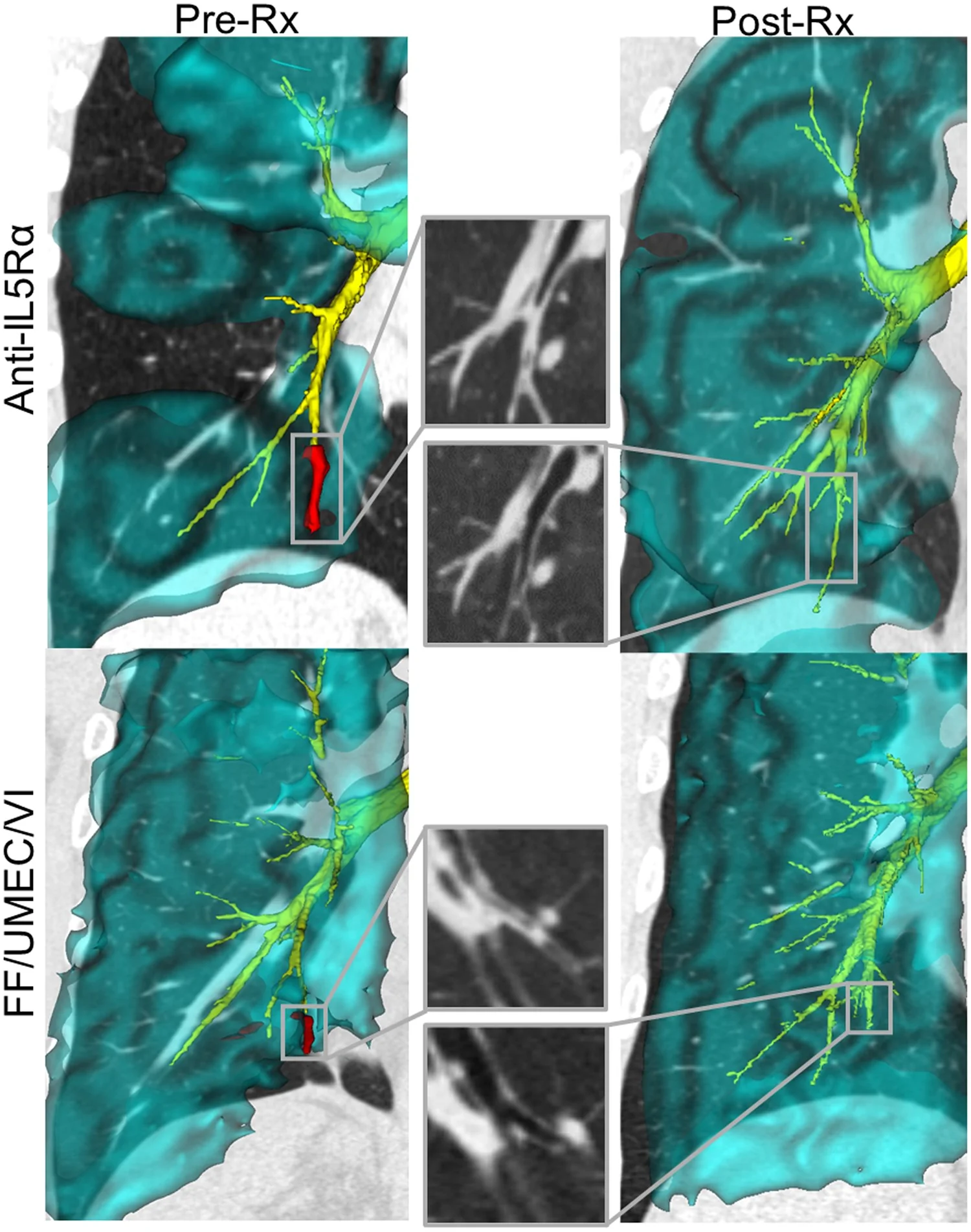
Demonstration of mucus plug resolution with treatment. Three-dimensional CT airway reconstructions (yellow) coregistered with ^129^Xe MRI ventilation (cyan) showing mucus plugs (red) before and after treatment. Left panel inset (Pre-Rx) shows a mucus plug (red) identified within a subsegmental airway, with magnified axial CT views demonstrating luminal occlusion. Right panel inset (Post-Rx) shows resolution of the mucus plug and improved visualization of distal airways. Image courtesy of the research team of Grace Parraga, PhD. Top panel, pre- and posttreatment ^129^Xe MRI ventilation coregistered with CT airway tree for a representative subject receiving anti–IL-5Rα therapy. Participant is a 20-year-old woman with Pre- and Post-Rx FEV_1_% predicted of 57% and 98%, respectively; Pre- and Post-Rx ACQ-6 score of 3.3 and 0.5, respectively; and Pre- and Post-Rx VDP of 34% and 9%, respectively. Bottom panel, Pre- and Post-Rx ^129^Xe MRI ventilation coregistered with CT airway tree for a representative subject receiving FF/UMEC/VI triple therapy. Participant is a 51-year-old man with Pre- and Post-Rx FEV_1_% predicted of 75% and 84%, respectively; Pre- and Post-Rx ACQ-6 score of 1.7 and 0.7, respectively; and Pre- and Post-Rx VDP of 5% and 4%, respectively. Abbreviations: ACQ, Asthma Control Questionnaire; CT, computed tomography; FF/UMEC/VI, fluticasone furoate/umeclidinium/vilanterol; MRI, magnetic resonance imaging; Post-Rx, posttreatment; Pre-Rx, pretreatment; VDP, ventilation defect percentage.

Importantly, interventional studies, as well as post hoc analyses of the VESTIGE and SARP-3 trials, agree that a decrease in mucus plug burden is associated with improved lung function. An example of cleared mucus plugs and associated changes in ventilation is displayed in Figure 4. Post hoc analysis of the VESTIGE trial demonstrated that dupilumab treatment was not only associated with a reduction in mucus plug segment score, but also that patients with decreasing mucus plug burden had the largest increase in FEV_1_, suggesting that treating mucus plugs may cause increased airflow in patients with moderate to severe asthma.^7^^,^^30^^,^^86^ Together, these clinical data, combined with pathologic and mechanistic data, contribute to an understanding of occluding mucus plugs as a dominant, potentially reversible contributor to airflow obstruction. In light of this combined evidence, many have embraced the presence of CT-detected mucus plugs as a treatable trait in pulmonary disease.^17^^,^^87-89^

## Scientific and clinical implications

While the existing research addressing CT-detected mucus plugs is compelling, it is not yet complete. CT detection of mucus plugs offers opportunities for both current clinical applications and future research to address critical gaps.

### Applying current evidence to clinical and scientific use

Existing evidence supports the current use of CT-detected mucus plugs for predicting clinical course, disease-specific phenotyping, and therapy response. In both prognostication^90^^,^^91^ and phenotyping,^62^^,^^92-94^ CT-detected mucus plug burden is ready for incorporation into clinical practice. To this end, we encourage radiologists to routinely report the presence or absence of mucus plugs in CT chest examinations of patients with asthma and COPD. We would also recommend describing the airway level at which they are identified, along with the lobes involved, which can be beneficial for assessing the extent and severity of mucus plugs in the absence of quantitative tools. If a prior chest CT is available at the time of interpretation, we recommend describing whether the mucus plugs are unchanged, increased, or decreased, as this will aid in understanding the impact of clinical treatments.

Data from published studies on asthma and COPD indicate that participants with mucus plugs involving ≥3 bronchopulmonary segments (mucus plug segment score of ≥3) are the population of greatest interest and serve as a cut-off threshold for clinical populations. A recent study, based on clinical and spirometry data, proposed an easy-to-use index termed SIMP to predict a CT MPS of ≥3.^95^ A lower burden of mucus plugs may also be useful for phenotyping and prognostication, as well as for future treatment efforts aimed at resolving mucus plugs, but the degree of benefit is less clear.

### Regulatory considerations

Quantitative imaging methods that measure clinically relevant attributes of pulmonary disease have the potential for regulatory use to promote innovation and efficiencies in clinical trials for the development of therapeutics. Available data suggest that imaging characterization of mucus plugs, both visual and quantitative, has potential as a prognostic, predictive, and/or response biomarker, but several uncertainties and data gaps remain to be addressed. The medical community must consider the incremental steps needed to qualify CT detection of mucus plugs as a biomarker with regulatory agencies. There are currently insufficient data to qualify mucus plugs as an intermediate study endpoint in therapeutic trials, but more modest contexts of use are possible. An initial step could be to first pursue qualification of visually scored mucus plugs in a limited context of use, which could subsequently expand in partnership with regulatory agencies into more fully automated quantitative methods of measuring airway mucus volume as additional data emerge. Additional features, such as mucus plug density, location, and length, are compelling and must continue to be explored even if not part of an initial regulatory submission.

To generate the data necessary to address open questions, the assessment of mucus plugging needs to be incorporated into clinical trials. Standardization of methodology and imaging acquisition is needed to facilitate the interpretation and integration of data across studies. Data on the responsiveness of mucus plugs to therapeutic interventions will be necessary to support regulatory use as a response biomarker for product labeling. Robust data from clinical trials that show consistent and strong evidence that the effect on mucus plugging predicts a specific clinical benefit will be necessary to support use as a validated surrogate endpoint. As data accumulate, our understanding of the use of imaging to assess mucus plugging for regulatory purposes will evolve. Routine use of CT for mucus plugging in clinical trials as a predictive, prognostic, or response biomarker is an important step forward.

### Current research gaps

Three additional areas of ongoing research need to be strengthened: (1) the reliability of quantitative detection, (2) the mechanisms by which mucus plugs contribute to poor outcomes and the potential therapeutic impact, and (3) the treatment effect of current therapies. While most current approaches to quantitative detection have demonstrated promising performance in single cohorts or within constrained imaging protocols, critical gaps remain in algorithm reproducibility and generalizability across scanners, reconstruction kernels, and radiation dose levels. Agreement among readers using different scanners and protocols has not yet been established. Furthermore, quantitative methods for identifying mucus plugs have not been widely validated against visual reads. These refinements will be necessary for standardized approaches toward use as a radiologic biomarker.

While extensive observational data has established the relationship between mucus plugs and poor outcomes, the exact mechanisms by which mucus plugs contribute to poor outcomes is still relatively unknown. Exploration of these mechanisms, including the contribution of mucus plug burden to cardiovascular, respiratory, and systemic pathology, would aid in understanding the expected therapeutic response to mucus plug treatment.

Given the established relationship between mucus plugs and poor outcomes, studies exploring therapeutic approaches to control the presence and extent of mucus plugs in COPD, asthma, and bronchiectasis are also sorely needed. Existing inhaled treatments, mucolytics, osmotically active agents, and mechanical treatments have not been robustly evaluated for effectiveness in reducing mucus plug burden. Further investigation should focus on the effects of existing therapies on mucus plug burden in COPD and asthma. Additionally, the role of mechanical clearance with bronchoscopy, chest physiotherapy, or direct administration of mucolytics should be further explored.

### Future directions

Phenotyping of mucus plugs is an important area of ongoing research. Current imaging-based definitions of mucus plugs primarily focus on fully occlusive plugs. However, accumulating evidence suggests that nonocclusive mucus, including mural-adherent mucus plaques, partial luminal narrowing, and mucus proximal to prematurely terminating airways, may represent an underrecognized but clinically meaningful phenotype. Additionally, the structure, shape, and longitudinal changes in mucus plugs may reflect different underlying biological processes. Furthermore, not all mucus plugs manifest the same inflammatory pattern; plugs have been identified with predominantly eosinophilic, neutrophilic, and lymphocytic patterns as well as bland patterns containing only macrophages.^10^^,^^28^^,^^96^ Notably, relationships among inflammation, microbiome profile, and metabolomic signature suggest novel therapeutic opportunities.^97^ Therefore, studies are needed to accurately identify the predominant mechanism responsible for plugging phenomena across individual disease states and plug types. While this review predominantly focuses upon mucus plugs within established disease states such as asthma and COPD, increasing attention is being paid to characterizing the early phase of chronic disease development.^98^^,^^99^ The role of mucus plugs in early disease development and formation is yet to be elucidated.

Novel mucolytic strategies are also currently under development, such as mucin disulfide bond–reducing agents that degrade mucus plugs faster and use lower administered concentrations of nebulized drug than *N*-acetyl cysteine.^100^ These agents may improve mucus dysfunction, enhance mucociliary clearance, and reduce airway hyperreactivity in preclinical models. Additional studies should focus on mucus hydration therapies to target the hyperconcentration of mucus that drives plug adhesion, including osmotically active solutes and pharmacologic manipulation of sodium absorption versus chloride secretion.^101^ Finally, interventions using advanced bronchoscopic approaches such as rheoplasty,^102^ cryotherapy, and specific lung denervation to modulate goblet cell hyperplasia are currently being explored.

## Conclusion

Mucus plugs contribute to deteriorations in lung function, respiratory symptoms, exacerbations, and poor clinical outcomes in both asthma and COPD. Advances in quantitative imaging represent an unprecedented opportunity to better define the presence and extent of mucus plugs as well as the response to treatment. The time has come to incorporate formal mucus plug assessment into both research outcomes and clinical care.

## Supplementary Material

aamag309_Supplementary_Data

## References

[1] Hogg JC , Macklem PT, Thurlbeck WM. Site and nature of airway obstruction in chronic obstructive lung disease. N Engl J Med. 1968;278:1355-1360. 10.1056/NEJM196806202782501 5650164

[2] McLean KH. The pathogenesis of pulmonary emphysema. Am J Med. 1958;25:62-74. 10.1016/0002-9343(58)90199-2 13559262

[3] Dunnill MS. The pathology of asthma, with special reference to changes in the bronchial mucosa. J Clin Pathol. 1960;13:27-33. 10.1136/jcp.13.1.27 13818688 PMC479992

[4] Huber HL , Koessler KK. The pathology of bronchial asthma. Arch Intern Med. 1922;30:689-760. 10.1001/archinte.1922.00110120002001

[5] Dunican EM , Elicker BM, Gierada DS, et al; National Heart Lung and Blood Institute (NHLBI) Severe Asthma Research Program (SARP). Mucus plugs in patients with asthma linked to eosinophilia and airflow obstruction. J Clin Invest. 2018;128:997-1009. 10.1172/JCI95693 29400693 PMC5824874

[6] Labaki WW , Martinez CH, Martinez FJ, et al The role of chest computed tomography in the evaluation and management of the patient with chronic obstructive pulmonary disease. Am J Respir Crit Care Med. 2017;196:1372-1379. 10.1164/rccm.201703-0451PP 28661698 PMC5736976

[7] Castro M , Papi A, Porsbjerg C, et al Effect of dupilumab on exhaled nitric oxide, mucus plugs, and functional respiratory imaging in patients with type 2 asthma (VESTIGE): a randomised, double-blind, placebo-controlled, phase 4 trial. Lancet Respir Med. 2025;13:208-220. 10.1016/S2213-2600(24)00362-X 39947221

[8] Diaz AA , Orejas JL, Grumley S, et al Airway-occluding mucus plugs and mortality in patients with chronic obstructive pulmonary disease. JAMA. 2023;329:1832-1839. 10.1001/jama.2023.2065 37210745 PMC10201404

[9] Okajima Y , Come CE, Nardelli P, et al Luminal plugging on chest CT Scan: association with lung function, quality of life, and COPD clinical phenotypes. Chest. 2020;158:121-130. 10.1016/j.chest.2019.12.046 32017932 PMC7339234

[10] Hill DB , Button B, Rubinstein M, Boucher RC. Physiology and pathophysiology of human airway mucus. Physiol Rev. 2022;102:1757-1836. 10.1152/physrev.00004.2021 35001665 PMC9665957

[11] Boucher RC. Muco-obstructive lung diseases. N Engl J Med. 2019;380:1941-1953. 10.1056/NEJMra1813799 31091375

[12] Thornton DJ , Sheehan JK. From mucins to mucus: toward a more coherent understanding of this essential barrier. Proc Am Thorac Soc. 2004;1:54-61. 10.1513/pats.2306016 16113413

[13] Fahy JV , Dickey BF. Airway mucus function and dysfunction. N Engl J Med. 2010;363:2233-2247. 10.1056/NEJMra0910061 21121836 PMC4048736

[14] Innes AL , Carrington SD, Thornton DJ, et al Ex vivo sputum analysis reveals impairment of protease-dependent mucus degradation by plasma proteins in acute asthma. Am J Respir Crit Care Med. 2009;180:203-210. 10.1164/rccm.200807-1056OC 19423716 PMC2724713

[15] Yuan S , Hollinger M, Lachowicz-Scroggins ME, et al Oxidation increases mucin polymer cross-links to stiffen airway mucus gels. Sci Transl Med. 2015;7:276ra27. 10.1126/scitranslmed.3010525 PMC4403633 25717100

[16] Patarin J , Ghiringhelli É, Darsy G, et al Rheological analysis of sputum from patients with chronic bronchial diseases. Sci Rep. 2020;10:15685. 10.1038/s41598-020-72672-6 32973305 PMC7518272

[17] Innes AL , Woodruff PG, Ferrando RE, et al Epithelial mucin stores are increased in the large airways of smokers with airflow obstruction. Chest. 2006;130:1102-1108. 10.1378/chest.130.4.1102 17035444

[18] Addante A , Raymond W, Gitlin I, et al A novel thiol-saccharide mucolytic for the treatment of muco-obstructive lung diseases. Eur Respir J. 2023;61:2202022. 10.1183/13993003.02022-2022 37080569 PMC10209473

[19] Liegeois MA , Braunreuther M, Charbit AR, et al Peroxidase-mediated mucin cross-linking drives pathologic mucus gel formation in IL-13–stimulated airway epithelial cells. *JCI Insight*. 2024;9:181024. 10.1172/jci.insight.181024 PMC11383604 38889046

[20] Randell SH , Boucher RC; University of North Carolina Virtual Lung Group. Effective mucus clearance is essential for respiratory health. Am J Respir Cell Mol Biol. 2006;35:20-28. 10.1165/rcmb.2006-0082SF 16528010 PMC2658694

[21] Esther CR , Muhlebach MS, Ehre C, et al Mucus accumulation in the lungs precedes structural changes and infection in children with cystic fibrosis. *Sci Transl Med*. 2019;11:eaav3488. 10.1126/scitranslmed.aav3488 30944166 PMC6566903

[22] Kesimer M , Ford AA, Ceppe A, et al Airway mucin concentration as a marker of chronic bronchitis. N Engl J Med. 2017;377:911-922. 10.1056/NEJMoa1701632 28877023 PMC5706541

[23] Tilley AE , Walters MS, Shaykhiev R, Crystal RG. Cilia dysfunction in lung disease. Annu Rev Physiol. 2015;77:379-406. 10.1146/annurev-physiol-021014-071931 25386990 PMC4465242

[24] Mikami Y , Grubb BR, Rogers TD, et al Chronic airway epithelial hypoxia exacerbates injury in muco-obstructive lung ­disease through mucus hyperconcentration. Sci Transl Med. 2023;15:eabo7728. 10.1126/scitranslmed.abo7728 37285404 PMC10664029

[25] Shao MXG , Nakanaga T, Nadel JA. Cigarette smoke induces MUC5AC mucin overproduction via tumor necrosis factor-α-converting enzyme in human airway epithelial (NCI-H292) cells. Am J Physiol Lung Cell Mol Physiol. 2004;287:L420-L427. 10.1152/ajplung.00019.2004 15121636

[26] Gensch E , Gallup M, Sucher A, et al Tobacco smoke control of mucin production in lung cells requires oxygen radicals AP-1 and JNK. J Biol Chem. 2004;279:39085-39093. 10.1074/jbc.M406866200 15262961

[27] Amatngalim GD , Vieira RP, Meiners S, Bartel S. Novel insights into the effects of cigarette smoke on the airway epithelial surface—lessons learned at the European Respiratory Society International Congress 2018 in Paris. J Thorac Dis. 2018;10:S2977-S2982. 10.21037/jtd.2018.08.17 30310684 PMC6174130

[28] Polverino F , Borgaonkar R, Cong C, et al Mucus plugs on CT and small airway pathology in COPD. *ERJ Open Res*. 2025;11:00546-2025. 10.1183/23120541.00546-2025 41367648 PMC12683592

[29] Liegeois MA , Hsieh A, Al-Fouadi M, et al Cellular and molecular features of asthma mucus plugs provide clues about their formation and persistence. *J Clin Invest*. 2025;135:e186889. 10.1172/JCI186889 40091838 PMC11910225

[30] Fahy JV. Progress in treating mucus plugs in asthma. Am J Respir Crit Care Med. 2026;212:202-204. 10.1164/rccm.202509-2140ED 41151010 PMC13254533

[31] Asakura T , Okuda K, Chen G, et al Proximal and distal bronchioles contribute to the pathogenesis of non-cystic fibrosis bronchiectasis (NCFB). Am J Respir Crit Care Med. 2024;209:374-389. 10.1164/rccm.202306-1093OC 38016030 PMC10878387

[32] Ramsey KA , Chen ACH, Radicioni G, et al Airway mucus hyperconcentration in non-cystic fibrosis bronchiectasis. Am J Respir Crit Care Med. 2020;201:661-670. 10.1164/rccm.201906-1219OC 31765597 PMC7068838

[33] Gilley SK , Stenbit AE, Pasek RC, et al Deletion of airway cilia results in noninflammatory bronchiectasis and hyperreactive airways. Am J Physiol Lung Cell Mol Physiol. 2014;306:L162-L169. 10.1152/ajplung.00095.2013 24213915 PMC3920206

[34] Fujisawa T , Chang MM-J, Velichko S, et al NF-κB mediates IL-1β- and IL-17A-induced MUC5B expression in airway epithelial cells. Am J Respir Cell Mol Biol. 2011;45:246-252. 10.1165/rcmb.2009-0313OC 20935193 PMC3175554

[35] Kuperman DA , Huang X, Koth LL, et al Direct effects of interleukin-13 on epithelial cells cause airway hyperreactivity and mucus overproduction in asthma. Nat Med. 2002;8:885-889. 10.1038/nm734 12091879

[36] Wang BX , Wheeler KM, Cady KC, et al Mucin glycans signal through the sensor kinase RetS to inhibit virulence-associated traits in *Pseudomonas aeruginosa*. Curr Biol. 2021;31:90-102.e7. 10.1016/j.cub.2020.09.088 33125866 PMC8759707

[37] Haley CL , Kruczek C, Qaisar U, Colmer-Hamood JA, Hamood AN. Mucin inhibits *Pseudomonas aeruginosa* biofilm formation by significantly enhancing twitching motility. Can J Microbiol. 2014;60:155-166. 10.1139/cjm-2013-0570 24588389 PMC4199588

[38] Huang BK , Elicker BM, Henry TS, et al Persistent mucus plugs in proximal airways are consequential for airflow limitation in asthma. *JCI Insight*. 2024;9:e174124. 10.1172/jci.insight.174124 PMC10967478 38127464

[39] Schworer SA , Murano H, Dang H, et al Airway epithelial heterogeneity and mucus plugging in asthmatic bronchioles. Am J Respir Crit Care Med. 2026;212:209-226. 10.1164/rccm.202409-1849OC 40986379 PMC12668797

[40] McIntosh MJ , Kooner HK, Eddy RL, et al CT mucus score and (129)Xe MRI ventilation defects after 2.5 years’ anti-IL-5Rα in eosinophilic asthma. Chest. 2023;164:27-38. 10.1016/j.chest.2023.02.009 36781102

[41] Mummy DG , Dunican EM, Carey KJ Jr, et al Mucus plugs in asthma at CT associated with regional ventilation defects at (3)He MRI. Radiology. 2022;303:184-190. 10.1148/radiol.2021204616 34931858 PMC8962781

[42] Wan ES , Borgaonkar R, Mettler S, et al Lower lobe mucus plug distribution and future respiratory exacerbations in tobacco-exposed individuals with and without COPD. *Eur Respir J*. 2026;67:0523-2025. 10.1183/13993003.00523-2025 PMC12859690 41309271

[43] Saetta M , Di Stefano A, Rosina C, Thiene G, Fabbri LM. Quantitative structural analysis of peripheral airways and arteries in sudden fatal asthma. Am Rev Respir Dis. 1991;143:138-143. 10.1164/ajrccm/143.1.138 1986670

[44] Rodriguez-Roisin R , Ballester E, Roca J, Torres A, Wagner PD. Mechanisms of hypoxemia in patients with status asthmaticus requiring mechanical ventilation. Am Rev Respir Dis. 1989;139:732-739. 10.1164/ajrccm/139.3.732 2923373

[45] Physical Activity Guidelines Advisory Committee. 2018 Physical Activity Guidelines Advisory Committee Scientific Report. US Department of Health and Human Services; 2018.

[46] Ceriana P , Vitacca M, Carlucci A, Paneroni M, Pisani L, Nava S. Changes of respiratory mechanics in COPD patients from stable state to acute exacerbations with respiratory failure. COPD. 2017;14:150-155. 10.1080/15412555.2016.1254173 27997251

[47] Dunican EM , Elicker BM, Henry T, et al Mucus plugs and emphysema in the pathophysiology of airflow obstruction and hypoxemia in smokers. Am J Respir Crit Care Med. 2021;203:957-968. 10.1164/rccm.202006-2248OC 33180550 PMC8048745

[48] Kawagoe Y , Permutt S, Fessler HE. Hyperinflation with intrinsic PEEP and respiratory muscle blood flow. J Appl Physiol (1985). 1994;77:2440-2448. 10.1152/jappl.1994.77.5.2440 7868467

[49] Vassaux C , Torre-Bouscoulet L, Zeineldine S, et al Effects of hyperinflation on the oxygen pulse as a marker of cardiac performance in COPD. Eur Respir J. 2008;32:1275-1282. 10.1183/09031936.00151707 18550609

[50] Freiberg DB , Colebatch HJ. Malignant asthma presenting as right heart failure. Med J Aust. 1987;147:90-92. 10.5694/j.1326-5377.1987.tb133269.x 3600457

[51] MacDonald MI , Shafuddin E, King PT, Chang CL, Bardin PG, Hancox RJ. Cardiac dysfunction during exacerbations of chronic obstructive pulmonary disease. Lancet Respir Med. 2016;4:138-148. 10.1016/S2213-2600(15)00509-3 26781000

[52] van der Veer T , Andrinopoulou E-R, Braunstahl G-J, et al Association between automatic AI-based quantification of airway-occlusive mucus plugs and all-cause mortality in patients with COPD. Thorax. 2025;80:105-108. 10.1136/thorax-2024-221928 39638548 PMC12052038

[53] Raut P , Chen Y, Taleb A, et al Validation of an artificial intelligence-based automated PRAGMA and mucus plugging algorithm in pediatric cystic fibrosis. J Cyst Fibros. 2025;24:970-978. 10.1016/j.jcf.2025.08.003 40841295

[54] Sonoda Y , Fukuda K, Matsuzaki H, et al A deep learning-based automated detection of mucus plugs in chest CT. ERJ Open Res. 2025;11:00377-02025. 10.1183/23120541.00377-2025 41367647 PMC12683589

[55] Souery WN , Billatos E, Elalami R, et al Mucus plugs–associated gene expression identifies pathophysiology shared with chronic bronchitis. Am J Respir Crit Care Med. 2025;212:253-267. 10.1164/rccm.202501-0262OC PMC12560677 41072469

[56] Kooner HK , Serajeddini H, Eddy RL, Yamashita C, Svenningsen S, Parraga G. Airway mucus in older people without chronic respiratory illness. Chest. 2024;166:429-432. 10.1016/j.chest.2024.04.023 38815621 PMC11443240

[57] Tran C , Singh GV, Haider E, et al Luminal mucus plugs are spatially associated with airway wall thickening in severe COPD and asthma: a single-centered, retrospective, observational study. Respir Med. 2022;202:106982. 10.1016/j.rmed.2022.106982 36116144

[58] Tanabe N , Matsumoto H, Hayashi Y, et al Mucus plug density and type 2 inflammation in asthma and/or chronic obstructive pulmonary disease: ultrahigh-resolution computed tomography study. Am J Respir Crit Care Med. 2025;211:2208-2211. 10.1164/rccm.202503-0576RL 40460341

[59] Wielpütz MO , Puderbach M, Kopp-Schneider A, et al Magnetic resonance imaging detects changes in structure and perfusion, and response to therapy in early cystic fibrosis lung disease. Am J Respir Crit Care Med. 2014;189:956-965. 10.1164/rccm.201309-1659OC 24564281

[60] Ringwald FG , Wucherpfennig L, Martynova A, et al Automated scoring of airway abnormalities and mucus plugging in chest magnetic resonance imaging of cystic fibrosis using artificial intelligence. Comput Struct Biotechnol J. 2025;28:442-453. 10.1016/j.csbj.2025.10.025 41209289 PMC12593636

[61] Tang M , Elicker BM, Henry T, et al Mucus plugs persist in asthma, and changes in mucus plugs associate with changes in airflow over time. Am J Respir Crit Care Med. 2022;205:1036-1045. 10.1164/rccm.202110-2265OC 35104436 PMC9851493

[62] Chan R , Duraikannu C, Lipworth B. Clinical associations of mucus plugging in moderate to severe asthma. J Allergy Clin Immunol Pract. 2023;11:195-199.e2. 10.1016/j.jaip.2022.09.008 36152990

[63] Tamura K , Shirai T, Hirai K, et al Mucus plugs and small airway dysfunction in asthma, COPD, and asthma-COPD overlap. Allergy Asthma Immunol Res. 2022;14:196-209. 10.4168/aair.2022.14.2.196 35255537 PMC8914605

[64] Svenningsen S , Haider E, Boylan C, et al CT and functional MRI to evaluate airway mucus in severe asthma. Chest. 2019;155:1178-1189. 10.1016/j.chest.2019.02.403 30910637

[65] Hsieh A , Vasilescu DM, Barker-Mulleder J, et al Mucus plugs correlate with small airway remodelling in asthma: a case-control study. Am J Respir Crit Care Med. 2025;212:227-240. 10.1164/rccm.202501-0310OC 40845271 PMC13234446

[66] Allinson JP , Hardy R, Donaldson GC, Shaheen SO, Kuh D, Wedzicha JA. The presence of chronic mucus hypersecretion across adult life in relation to chronic obstructive pulmonary disease development. Am J Respir Crit Care Med. 2016;193:662-672. 10.1164/rccm.201511-2210OC 26695373 PMC4824943

[67] Hogg JC , Chu F, Utokaparch S, et al The nature of small-airway obstruction in chronic obstructive pulmonary disease. N Engl J Med. 2004;350:2645-2653. 10.1056/NEJMoa032158 15215480

[68] Hogg JC , Chu FSF, Tan WC, et al Survival after lung volume reduction in chronic obstructive pulmonary disease: insights from small airway pathology. Am J Respir Crit Care Med. 2007;176:454-459. 10.1164/rccm.200612-1772OC 17556723 PMC1976540

[69] Wan E , Yen A, Elalami R, et al Airway mucus plugs on chest computed tomography are associated with exacerbations in COPD. Am J Respir Crit Care Med. 2024;211:814-822. 10.1164/rccm.202403-0632OC PMC12091021 39470402

[70] Borgaonkar R , Iturrioz-Campo M, Nardelli P, et al Mucus plugs and exacerbations in tobacco-exposed people with normal and impaired spirometry. Ann Am Thorac Soc. 2026;23:318-321. 10.1093/annalsats/aaoaf001 41759068 PMC13019521

[71] Mettler SK , Nath HP, Grumley S, et al Silent airway mucus plugs in COPD and clinical implications. Chest. 2024;166:1010-1019. 10.1016/j.chest.2023.11.033 38013161 PMC11562650

[72] Li X , Feng S, Yang Y, et al Association between airway mucus plugs and risk of moderate-to-severe exacerbations in patients with COPD: results from a Chinese prospective cohort study. Chest. 2025;168:627-638. 10.1016/j.chest.2025.03.026 40210091

[73] Mettler SK , Nardelli P, Campo MI, et al; COPDGene Site Investigators. Longitudinal changes in airway mucus plugs and FEV(1) in COPD. N Engl J Med. 2025;392:1973-1975. 10.1056/NEJMc2502456 40367384 PMC12356501

[74] Olanipekun TT , Mettler S, Sharma N, et al Respiratory oscillometry in COPD patients with airway mucus plugs: insights from the ECLIPSE study. Am J Respir Crit Care Med. 2026;212:2079-2082. 10.1093/ajrccm/aamag244 42184284 PMC13274801

[75] Tanabe N , Shimizu K, Shima H, et al Computed tomography mucus plugs and airway tree structure in patients with chronic obstructive pulmonary disease: associations with airflow limitation, health-related independence and mortality. Respirology. 2024;29:951-961. 10.1111/resp.14776 38924669

[76] Kim SJ , Park H, Lee HJ, et al Mucus plug and lung cancer incidence in patients with COPD. Sci Rep. 2025;15:30193. 10.1038/s41598-025-13501-6 40825984 PMC12361539

[77] Mettler SK , Sonavane S, Grumley S, et al Airway-occluding mucus plugs and cause-specific mortality in chronic obstructive pulmonary disease. Am J Respir Crit Care Med. 2024;209:1508-1510. 10.1164/rccm.202401-0121LE 38771048 PMC11208961

[78] Svenningsen S , Kjarsgaard M, Haider E, et al Effects of dupilumab on mucus plugging and ventilation defects in patients with moderate-to-severe asthma: a randomized, double-blind, placebo-controlled trial. Am J Respir Crit Care Med. 2023;208:995-997. 10.1164/rccm.202306-1102LE 37603097

[79] Sakai N , Koya T, Murai Y, et al Effect of benralizumab on mucus plugs in severe eosinophilic asthma. Int Arch Allergy Immunol. 2023;184:783-791. 10.1159/000530392 37231966

[80] McIntosh MJ , Kooner HK, Eddy RL, et al Asthma control, airway mucus, and (129)Xe MRI ventilation after a single benralizumab dose. Chest. 2022;162:520-533. 10.1016/j.chest.2022.03.003 35283104

[81] Campisi R , Nolasco S, Bonsignore M, et al Effectiveness of mepolizumab on mucus plug reduction and clinical outcomes in severe eosinophilic asthma: a prospective observational study. J Allergy Clin Immunol Pract. 2026;14:415-417. 10.1016/j.jaip.2025.10.015 42633588

[82] Hara Y , Tanabe N, Marumo S, et al Clinical impact of mepolizumab on airway mucus plugs in patients with severe asthma. J Asthma Allergy. 2025;18:1713-1725. 10.2147/JAA.S551901 41399491 PMC12702269

[83] Nordenmark LH , Hellqvist Å, Emson C, et al Tezepelumab and mucus plugs in patients with moderate-to-severe asthma. NEJM Evid. 2023;2:EVIDoa2300135. 10.1056/EVIDoa2300135 38320181

[84] Singh D , Guller P, Reid F, et al A phase 2a trial of the IL-33 monoclonal antibody tozorakimab in patients with COPD: Frontier-4. Eur Respir J. 2025;66:2402231. 10.1183/13993003.02231-2024 40154559 PMC12256803

[85] Mozaffaripour A , Tcherner S, Durom E, et al Airway mucus and (129)Xe MRI ventilation after single inhaler triple therapy in asthma. ERJ Open Res. 2025;11:01333-2024. 10.1183/23120541.01333-2024 41089567 PMC12517039

[86] Porsbjerg C , Dunican EM, Lugogo NL, et al Effect of dupilumab on mucus burden in patients with moderate-to-severe asthma: the VESTIGE trial. Am J Respir Crit Care Med. 2026;212:241-252. 10.1164/rccm.202410-1894OC 41145399 PMC13051685

[87] Chaudhary MFA , Bhatt SP. Imaging endpoints for biologic therapy in chronic obstructive pulmonary disease. Br J Radiol. 2025:tqaf179. 10.1093/bjr/tqaf179 PMC13389070 40742322

[88] Kirby M , Parraga G. CT evidence of airway luminal plugging in COPD: a new plug for targeted treatment? Chest. 2020;158:7-8. 10.1016/j.chest.2020.02.005 32654731

[89] Tanabe N. Airway mucus plugs in COPD: a potential treatable trait. Respirology. 2026;31:29-31. 10.1002/resp.70169 41266930

[90] Celli BR , Fabbri LM, Yohannes AM, et al A person-centred clinical approach to the multimorbid patient with COPD. Eur J Intern Med. 2025;140:106424. 10.1016/j.ejim.2025.07.020 40803921

[91] Singh D , Han MK, Bhatt SP, et al Is disease stability an attainable chronic obstructive pulmonary disease treatment goal? Am J Respir Crit Care Med. 2025;211:452-463. 10.1164/rccm.202406-1254CI 39680953 PMC11936119

[92] Diaz AA , Grumley S, Yen A, et al Eosinophils, mucus plugs and clinical outcomes: findings from two COPD cohorts. Eur Respir J. 2024;64:2401005. 10.1183/13993003.01005-2024 39572219 PMC13200003

[93] Götschke J , Walter J, Leuschner G, et al Mucus plug score predicts clinical and pulmonary function response to biologic therapy in patients with severe asthma. J Allergy Clin Immunol Pract. 2025;13:1110-1122.e1. 10.1016/j.jaip.2025.01.010 39826645

[94] Chan R , Lipworth B, Cottini M, et al Mucus plugging as a prognostic indicator for biologic treatment response in severe asthma. J Allergy Clin Immunol Pract. 2025;13:1123-1124. 10.1016/j.jaip.2025.01.022 40340084

[95] Wang W , Borgaonkar R, Mettler SK, et al A simple index for predicting mucus plugs in patients with COPD. Am J Respir Crit Care Med. 2025;212:277-280. 10.1164/rccm.202503-0563RL PMC12831125 40961266

[96] Ueki S , Dunican E, Diaz AA. Mucus plugs and eosinophilic inflammation: expanding the paradigm to COPD. J Allergy Clin Immunol Pract. 2026;14:575-582. 10.1016/j.jaip.2025.11.028 41317963 PMC12834202

[97] Tanabe N , Matsumoto H, Morimoto C, Hirai T. Sputum short-chain fatty acids, microbiome, inflammation, and mucus plugging in obstructive airway disease. J Allergy Clin Immunol. 2025;155:1675-1680. 10.1016/j.jaci.2025.01.031 39914553

[98] Ritchie AI , Donaldson GC, Hoffman EA, et al; British Early COPD Network (BEACON) Cohort Investigators. Structural predictors of lung function decline in young smokers with normal spirometry. Am J Respir Crit Care Med. 2024;209:1208-1218. 10.1164/rccm.202307-1203OC 38175920 PMC11146542

[99] Martinez FJ , Han MK, Allinson JP, et al At the root: defining and halting progression of early chronic obstructive pulmonary disease. Am J Respir Crit Care Med. 2018;197:1540-1551. 10.1164/rccm.201710-2028PP 29406779 PMC6006401

[100] Morgan LE , Jaramillo AM, Shenoy SK, et al Disulfide disruption reverses mucus dysfunction in allergic airway disease. Nat Commun. 2021;12:249. 10.1038/s41467-020-20499-0 33431872 PMC7801631

[101] Mall MA , Danahay H, Boucher RC. Emerging concepts and therapies for mucoobstructive lung disease. Ann Am Thorac Soc. 2018;15:S216-S226. 10.1513/AnnalsATS.201806-368AW 30431343 PMC6322026

[102] Krimsky WS , Mammarappallil JG, Kim V, et al Airway mucus plugging in chronic bronchitis and the impact of bronchial rheoplasty. Chest. 2026;169:73-83. 10.1016/j.chest.2025.06.022 40571054

